# New Method and Portable Measurement Device for the Calibration of Industrial Robots

**DOI:** 10.3390/s20205919

**Published:** 2020-10-20

**Authors:** Caglar Icli, Oleksandr Stepanenko, Ilian Bonev

**Affiliations:** École de Technologie Supérieure, Montreal, QC H3C 1K3, Canada; caglar.icli.1@ens.etsmtl.ca (C.I.); ol.stepanenko@icloud.com (O.S.)

**Keywords:** precision, robot calibration, robot accuracy, autonomous calibration, closed-loop calibration, self-calibration

## Abstract

This paper presents an automated calibration method for industrial robots, based on the use of (1) a novel, low-cost, wireless, 3D measuring device mounted on the robot end-effector and (2) a portable 3D ball artifact fixed with respect to the robot base. The new device, called TriCal, is essentially a fixture holding three digital indicators (plunger style), the axes of which are orthogonal and intersect at one point, considered to be the robot tool center point (TCP). The artifact contains four 1-inch datum balls, each mounted on a stem, with precisely known relative positions measured on a Coordinate Measuring Machine (CMM). The measurement procedure with the TriCal is fully automated and consists of the robot moving its end-effector in such as a way as to perfectly align its TCP with the center of each of the four datum balls, with multiple end-effector orientations. The calibration method and hardware were tested on a six-axis industrial robot (KUKA KR6 R700 sixx). The calibration model included all kinematic and joint stiffness parameters, which were identified using the least-squares method. The efficiency of the new calibration system was validated by measuring the accuracy of the robot after calibration in 500 nearly random end-effector poses using a laser tracker. The same validation was performed after the robot was calibrated using measurements from the laser tracker only. Results show that both measurement methods lead to similar accuracy improvements, with the TriCal yielding maximum position errors of 0.624 mm and mean position errors of 0.326 mm.

## 1. Introduction

The difference between robot repeatability and robot accuracy is well known in academia, but industrial clients of robot manufacturers often confound the two concepts, based on the personal experience of the third author, who is also cofounder of Mecademic, a manufacturer of compact, high-precision industrial robots, and instigator of RoboDK, a software for offline programming and calibration of industrial robots. Yet, while the position repeatability of most six-axis industrial robots is 0.1 mm or better, even the smallest and most precise robot arms such those from Mecademic can make positioning errors of more than 1 mm, unless calibrated.

Robot calibration is the process of replacing the nominal mathematical model of a robot with another set of equations, usually much more complex (e.g., assuming non-zero elasticities for the joints), which describes with less error the relationship between the joint values and the end-effector pose of the real robot [1,2]. To identify the parameters of the new mathematical model (lengths, angles, elasticities, etc.), the full or partial pose of the robot end-effector is measured for various sets of robot joint values (e.g., a hundred, random, or specially selected). The results are then fed to an optimization algorithm that identifies the parameters that minimize the errors between the actual pose measurements and the ones calculated by using the new mathematical model.

Ideally, the calibration must be made by the robot manufacturer, who has suitable installations involving high-accuracy measurement devices, such as an optical tracker, a laser tracker, or even a Coordinate Measuring Machine (CMM) [3]. The main advantage of in-house calibration, however, is that the new mathematical model is embedded directly in the robot controller. Thus, almost no extra steps or knowledge are required when using an in-house calibrated robot (in comparison to using a non-calibrated robot).

Unfortunately, not all clients realize immediately that they need calibration. Indeed, in applications that require offline programming, such as material removal, the need for calibration might be obvious. In other applications, such as inspection, calibration might be avoided but only by spending hours of tuning the robot path, each time a new part shape is to be inspected. Furthermore, while some manufacturers do offer on-site calibration services, most have to rent a laser tracker from a local provider of metrology services. This is why, many researchers, both from academia and industry, have worked on the development of portable, low-cost measurements tools for on-site robot calibration.

Various measurement tools have been implemented into calibration experimentations throughout time. For robot users, eye-in-hand devices such as touch probes [4], optical sensors [5], and ballbars [6] tend to be more affordable and easier to implement in comparison to external measurement devices such as laser trackers [7,8,9], mechanical CMMs [10], optical CMMs [11], theodolites [12], and measurement arms [13]. However, the effectiveness of eye-in-hand devices is still challenged when considering the comparison of the end-result accuracy obtained through external devices. 

Perhaps the simplest and oldest, low-cost measurement strategy in robot calibration is to constrain the position of the robot’s tool-center-point (TCP), vary the orientation of the robot end-effector, and record the corresponding robot joint values [14]. Instead of manually constraining the robot TCP to a mechanical coupling (e.g., a ball in a socket), in [15], we explored the embedded force-torque sensors in some collaborative robot arms, by mounting a special kinematic fixture and automatically performing the coupling using force control. For all other robots, we proposed in [16] a measuring device used to automatically drive the robot TCP to coincide with the center of a datum ball. This seemingly minor difference is in fact a significant improvement since no frequent manual back-and-forth jogging or force control capability is required and the method can be fully automated.

On the one hand, instruments similar to the device proposed in [16] already exist, namely ROSY [17] by Teconsult GmbH, Germany (based on two cameras), Laser LAB [18] by Wiest AG, Germany (based on five laser sensors) and ZIS Calibration Unit [19] from ZIS Industrietechnik GmbH, Germany (based on a 3-degree-of-freedom mechanism requiring a physical contact). However, not only is the accuracy of these three devices relatively poor (as low as 0.1 mm) but they are used as error measurement devices rather than replacements of a position’s physical constraint. This means that the robot’s TCP is never exactly at the center of the tool tip, but as far away as 10 mm. While this offset is taken into account in the calibration, it is not measured with high accuracy.

On the other hand, far more accurate devices based on the same principle have been used for calibrating machine tools. Some researchers have proposed a device called the R-Test [20], which uses three analog linear probes and has an uncertainty of 1.7 µm for a measurement range of less than 0.5 mm. A very similar device has also been proposed in [21] but using four probes. More recently, researchers have proposed another similar device based on three Keyence laser displacement sensors and cite multiple other recent research developments on the design of such high-accuracy 3D sensors [22]. Finally, the company IBS Precision Engineering (The Netherlands) offers the Trinity contact-free probe, which can measure the offset of the center of a special datum sphere (expensive and fragile) within a range of up to 3.5 mm (still too small for robotics) and with an accuracy of less than 0.001 mm. Unfortunately, all these devices are relatively expensive (the Trinity costs about USD 15,000), because they need to be built with great accuracy and calibrated before use. For example, we could not find a way to identify the measurement reference frame of our Trinity probe with respect to its fixture, which is simply a cylindrical shaft. We believe that these are excellent measuring solution for machine tools, but somewhat inadequate for industrial robots, at least in factory settings.

**Figure 1:**
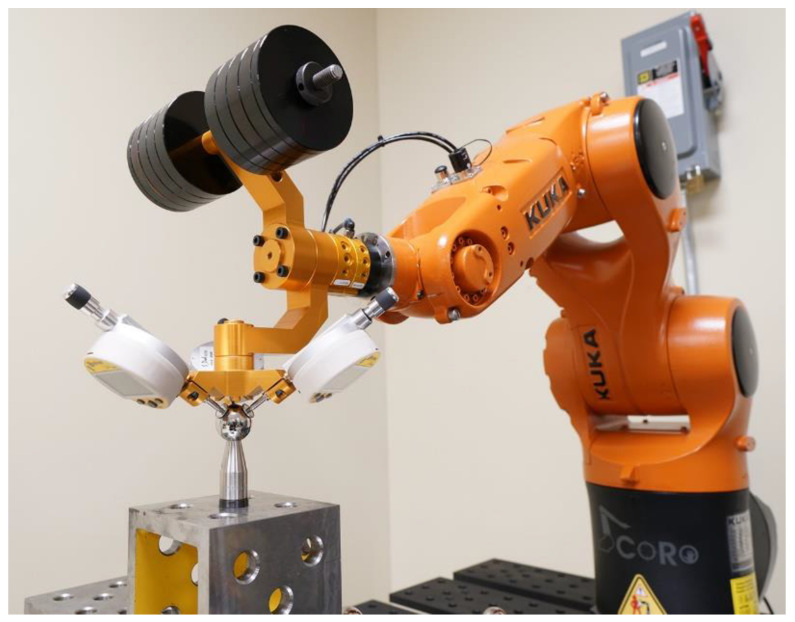
TriCal measuring device installed on a KUKA KR 6 R700 sixx industrial robot.

Most importantly, none of the device mentioned in the preceding two paragraphs are used to constraint the TCP to a given position. In this paper, we present a new device and method based on the same idea as the one presented in [16] but with several major improvements:

− The new device (Figure 1) is more practical and ready for use in industrial settings. It does not need an additional calibrator plate for TCP initialization and provides the possibility to use additional weights.
− The new device is also more affordable and easier to service (several prototypes, excluding the digital indicators, have been sold to industry at only USD 2000 each, so the total cost of the device is less than USD 5000).
− Finally, we show that the new device can be almost as effective in robot calibration as a laser tracker, by reducing the maximum position errors of the robot to values only 15% higher than those in the case of a laser tracker.

In the remainder of this paper, we present the measurement device, the 3D ball artifact and the communication setup in Section 2, and then describe the measurement procedure in Section 3. Next, the accuracy of the measurement process is analysed in Section 4. Then, the robot modelling and parameter identification method are described in Section 5. The identified parameters and validation results are presented in Section 6. Finally, conclusions are made in Section 7.

## 2. Description of the New Calibration System

Figure 1 shows the new measurement device, called TriCal, and part of the experimental setup involving the 3D ball artifact (partially visible) and a six-axis KUKA KR6 R700 sixx industrial robot.

### 2.1. The TriCal Measurement Device

**Figure 2:**
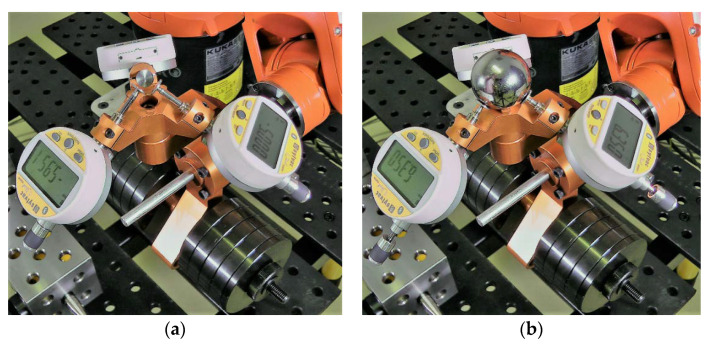
TriCal during mastering with the 1.5-in master ball. (**a**) TriCal before mastering. (**b**) TriCal after mastering.

**Figure 3:**
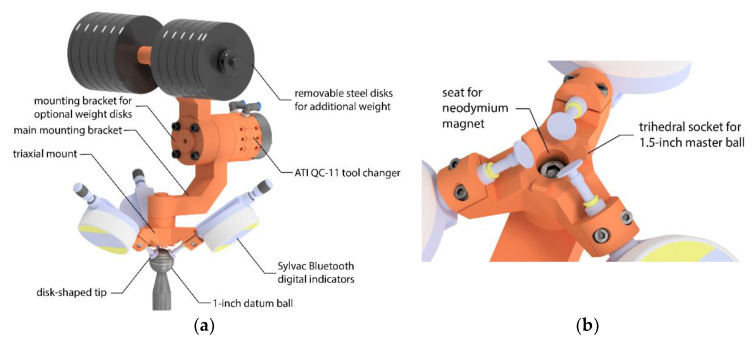
Description of the TriCal device: (**a**) overall view of TriCal; (**b**) close-up of triaxial mount.

The main part of TriCal is an aluminum triaxial mount with three Ø8-mm cylindrical channels with clamps, seen clearly in Figure 2 and Figure 3. The axes of these channels are orthogonal and intersect at one point, which will be defined as the robot tool center point (TCP). Indeed, the tool reference frame that will be defined in the robot is along the axes of these channels. A Sylvac S_Dial WORK NANO digital indicator is fixed to each of the three channels. A disk-shaped tip is attached to each digital indicator, instead of the typical ball tip, providing a flat contact surface, orthogonal to the axis of measurement. Thus, each indicator measures exactly the *x*, *y*, or *z* coordinate of the center of a ball with respect to the tool reference frame.

A major improvement in our new TriCal is the use of a *master ball* for mastering the digital indicators and defining the TCP (Figure 2a) that is larger than the *datum balls* (the precision balls of the 3D ball artifact). The use of this bigger master ball eliminates the need for a large kinematic platform that holds the master ball in the first version of TriCal [16]. Instead, a kinematic mount in the form of a trihedral socket is directly embedded in the center of the triaxial mount (Figure 2b and Figure 3b). When the master ball is placed in the socket (Figure 2a), the center of the ball defines the TCP and allows all digital indicators to be mastered. Furthermore, a removable rare-Earth magnet (not shown in Figure 2) can be placed in the socket, thus restraining the ball when needed. This magnet is only used (1) when measuring with a CMM the coordinates of the TCP with respect to the mounting surface of TriCal, by probing the master ball, and (2) for attaching an SMR (spherically-mounted reflector) during measurements with a laser tracker. During mastering, it suffices to orient TriCal upwards and the magnet is not needed; the force of gravity acting on the master ball is much larger than the equivalent of the forces applied by the three digital indicators.

Since the measurement range of each indicator is 12.5 mm, the radius of the master ball must be about 6 mm larger than the radius of the datum balls that are to be probed (Figure 1), to allow a symmetric measurement range for the complete TriCal device. Datum balls are readily available in sizes of 0.5 in, 0.75 in, and 1 in. As for the diameter of the master ball, we want to be able to validate our results with a laser tracker and spherically-mounted reflectors (SMRs) come only in diameters of 0.5 in, 0.875 in, and 1.5 in, with the smallest and largest diameter being the most popular. Indeed, as mentioned in the previous paragraph, during validation, we will place an SMR in the trihedral socket for the master ball, so both balls must have the same diameter. Therefore, we choose 1.5 in for the diameter of the master ball (and of the SMR) and 1.0 in for the diameter of the datum balls, which gives a difference in their radii of 6.350 mm.

The placement of the digital indicators along the cylindrical channels is extremely important in order to allow optimal use of the 12.5-mm measurement range of each indicator. This placement could have been restrained by implementing a physical stopper along the channels of the triaxial mount, but we chose not to do so, to allow for more flexibility (e.g., potentially use other digital indicators). Since we are going to use 1-in datum balls for the ball artifact, the indicators must be placed so that when a 1-in ball is centered at the TCP, the indicators are retracted halfway, i.e., approximately 6 mm. Since we want the indicators to display “0” in the latter situation, when the master 1.5-in ball is placed in the socket, each indicator must be set to display 0.25 in, i.e., 6.350 mm. This is called the mastering (Figure 2).

To know that each indicator is properly positioned along its axis, after the mastering, when you remove the master ball, each indicator must display −6.150 mm (6.350–12.500). In practice, however, this would require too many trials and errors. Furthermore, the 11.5 mm diameter of the disk-shaped tips provides another limitation. In order to have a plane-sphere contact, the center of the datum ball must not be farther than 5.750 mm from the axes of any of the indicators during the first contact. In other words, it is safer to assume that even when properly assembled, TriCal can only be used to measure the relative position of 1-in precision balls centered as far as 5 mm away from the TCP of TriCal. 

The remaining elements of TriCal (see Figure 1 and Figure 2a) are an aluminum mounting bracket for the triaxial mount that ends with a QC-11 tool changer from ATI, and another aluminum mounting bracket for optional steel weight disks. Thus, the total weight of the TriCal can range from 1.3 kg to 7 kg.

### 2.2. The 3D Ball Artifact

The 3D ball artifact is essentially a very rigid fixture with several 1-in datum balls positioned at precisely known locations in space (not just in one plane). Obviously, the more the datum balls, and the greater their distribution in the workspace of the robot, the better the performance of the calibration. However, for the proposed calibration system to be of any practical value, the artifact must be relatively portable and compact, as it must be carried by an operator and fit both on a medium-size CMM and in an existing robot cell.

**Figure 4:**
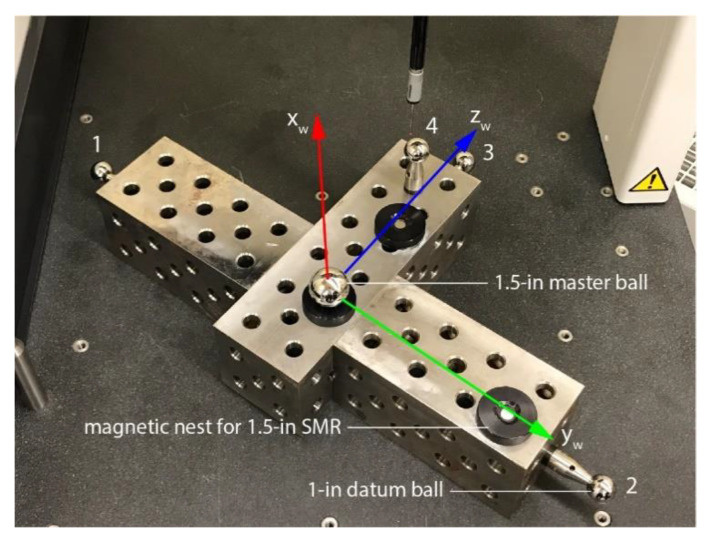
Characterizing the 3D ball artifact on a Coordinate Measuring Machine (CMM).

**Figure 5:**
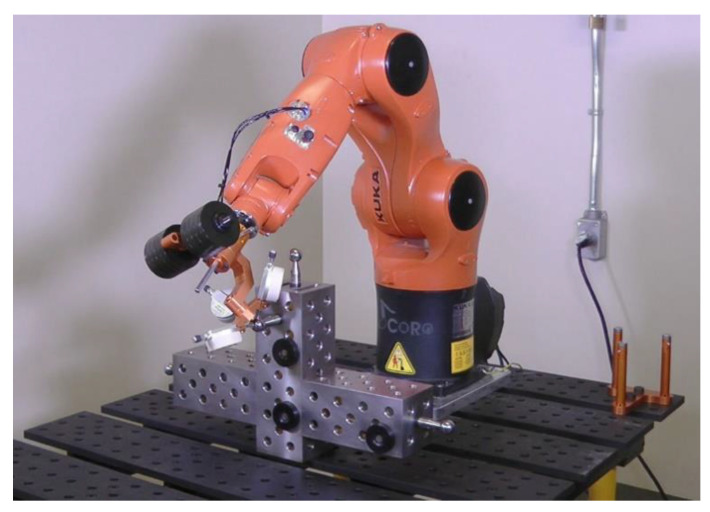
TriCal and the 3D ball artifact fixed, during measurements.

Since we use extensively the BuildPro collection of modular fixtures and tables from Valtra, which allow precise positioning, we chose to employ BuildPro heavy-duty riser blocks for the body of the artifact. Specifically, we selected one 7.8-kg and two 5.7-kg riser blocks and arranged them as shown in Figure 4 and Figure 5 to allow a maximum horizontal distance between two of the datum balls, and maximum height for the top datum ball. The vertical location of the two short riser blocks and of the fourth datum ball were selected through discretization and simulation in RoboDK [20], in order to allow a wide range of measurement orientations. As for the datum balls, these are manufactured by Micro Surface Engineering. The complete artifact weighs approximately 20 kg. 

For validation purposes, three magnetic nests for 1.5-in SMRs are also attached to the artifact. Their centers define the world reference frame, *x_w_y_w_z_w_*, as shown in Figure 4. The locations of the four datum balls with respect to that reference frame are then measured on a Mitutoyo CRYSTA-Apex C 544 CMM and are presented in Table 1.

### 2.3. Communication System

An extremely important advantage of TriCal is that the device is wireless, thanks to the embedded Bluetooth technology in the Sylvac digital indicators. The Bluetooth signals coming from each indicator are converted to virtual serial ports with the use of Sylvac’s V-MUX (Virtual Multiplexer) software in a PC. The PC then communicates with the robot via Ethernet. As we will describe in the following section, the main measurement procedure (the so-called auto-centering) essentially consists of moving the robot end-effector iteratively until all digital indicators measure (almost) zero displacement.

## 3. Measurement Procedure

During the measurement phase, the robot must probe each of the four datum balls with different end-effector orientations. Each probing of a datum ball is essentially an automated procedure for bringing the robot TCP at the center of the datum ball.

Before the measurement phase, the digital indicators are mastered with the 1.5-in master ball, placed manually onto the TriCal nest (Figure 2). Once the master ball in place, each indicator is set to 6.350 mm.

As already mentioned, it is crucial to measure on a CMM the axes of the cylindrical channels and the center of the master ball while in the trihedral socket, all with respect to the tool changer. Then, a tool reference frame is defined such that its center coincides with the center of the master ball and its axes coincide with the axes of the channels. Of course, since the axes are not perfectly orthogonal and concurrent, we used a 3D metrology software (PolyWorks) to find the best fit.

Once the tool reference frame is correctly defined, it is easy to jog the robot along the axes of the three digital indicators. After characterization (Figure 4), the artifact is placed in the robot cell in its prescribed location (Figure 5). In our case, since we use a BuldingPro welding table and fixtures that have precision holes equally spaced with tolerance of about 40 μm and surfaces with flatness of about 0.3 mm per 1 m, we know the positions of the datum balls with respect to the robot’s base frame with good accuracy (better than 1 mm). Furthermore, we have precise knowledge of the robot tool reference frame as it was measured on a CMM. Finally, a robot like the one used in our study typically has position errors that are less than 4 mm, before calibration. Therefore, even without calibration, the robot can bring its TCP well within the 5-mm measurement range of TriCal from the center of each datum ball. Thus, there is no need for manually teaching the positions of the datum balls and the whole measurement sequence can be programmed off-line.

Once the TriCal is positioned onto a datum ball using only the inherent position accuracy of the non-calibrated robot, an automated centering sequence is launched. Essentially, the measurements from the indicators are read in the PC and a linear motion command with the respective displacements relative to the tool reference frame is sent to the robot. The sequence is repeated several times, until all three indicators read displacements of less than 3 μm. Then, the robot joint values are retrieved in the PC and recorded. The auto-centering sequence lasts 30 s on the average.

The robot then backs the TriCal away, moves it to a different approach pose, then brings it to the datum ball with a different orientation, and finally the auto-centering procedure is launched again. Each of the four datum balls is probed with several different orientations.

**Figure 6:**
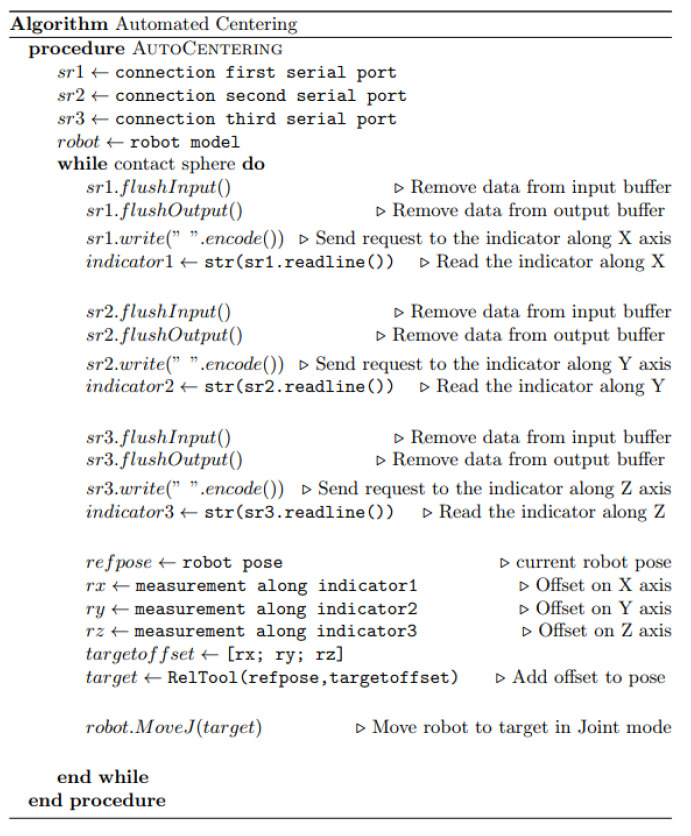
Automated centering algorithm.

The complete 3D environment was modeled in RoboDK [23] and the whole measurement procedure was implemented in the same software. Thus, 3D interferences were accounted for and avoided. Python scripts were written for interfacing with the indicators, for the auto-centering procedure (Figure 6), and for the complete measurement sequence. Note that RoboDK already comes with the necessary “plugins” to control a KUKA robot over Ethernet. Thus, the whole measurement procedure is fully automated and executed from RoboDK, except for the mastering of TriCal.

The whole procedure is summarized in Table 2.

## 4. Measurement Accuracy

In this section, we will estimate the accuracy of the complete system and measurement procedure. However, since the rated position repeatability of the robot used (or any similar small robot with a payload between 1.3 kg and 7 kg) is 30 μm, there is no need for complex analyses—a quick verification shows that our measurement accuracy is several times better than 30 μm. A summary of the validation is given in Table 3.

The sphericity and diameter tolerance of the 1-in datum balls and of the 1.5-in master ball used in this work are ±1.27 μm. The accuracy of the digital indicators is 1.8 μm. Finally, the Mitutoyo CRYSTA-Apex C 544 CMM used for characterization of TriCal has an uncertainty of 1.9 μm.

**Figure 7:**
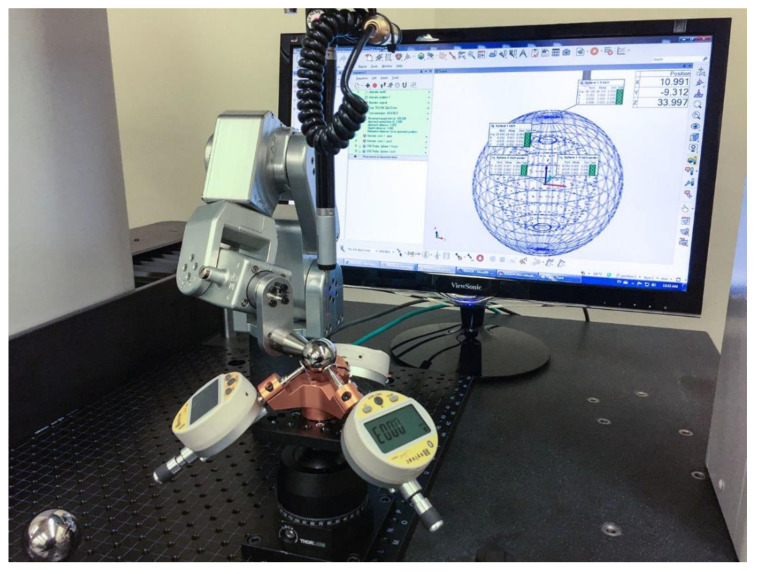
Setup for measuring the precision of TriCal.

If we were to use TriCal as a 3D measurement device, i.e., for measuring the precise offset of the center a 1-in datum ball with respect to the tool reference frame, then we must also measure the orthogonality of the three channels in the tri-axial mount of TriCal, or more importantly the orthogonality of the planar surfaces of the three disk-shaped tips, which we did. However, recall that we do not need TriCal to be accurate; we only need it be precise. Essentially, we need that when TriCal is centered over a 1-in datum ball such that all three indicators display “0.000”, the center of that datum ball is extremely close to the TCP, which is the center of the 1.5-in master ball during mastering. Therefore, we built the setup shown in Figure 7 and performed the following measurements on a CMM.

We first position the 1-5-in master ball in the trihedral socket of TriCal and master all three digital indicators (i.e., set them to 6.350 mm). Then, we measure the position of the master ball with the CMM. This is the actual TCP of TriCal. (We used a similar setup to measure that same TCP but with respect to the tool changer of the complete TriCal assembly.) We then removed the master ball and mounted one of the datum balls on a very small and highly precise robot arm—Mecademic’s Meca500 [24]. We then used another auto-centering procedure to move the datum ball until all indicators shows a maximum displacement of ±3 μm. We then measured the datum ball with the CMM. We repeated the last phase three times (i.e., measured the datum ball after three auto-centering sequences). The maximum deviation measured between the positions of the datum ball and the position of the master ball was about 2 μm.

Even if we consider the errors in the positions of the datum balls on the artifact (Figure 4) due to the 1.9 μm measurement uncertainty and the minor deflections of the artifact, our measurement procedure is clearly several times more accurate than the repeatability of the robot itself. In fact, it is even more accurate than a measurement with a laser tracker.

## 5. Robot Modeling and Parameter Identification

The robot kinematic parameters are modeled according to the Modified Denavit–Hartenberg (MDH) convention [25]. An additional parameter, $\beta_{i-1}$, defines the skewness between the axes of the second and third joint [26]. The robot nominal MDH parameters are presented in Table 4.

The homogeneous matrix transformation for each joint frame is calculated as,

(1) $$T_{i}^{i-1}=R_{x}(\alpha_{i-1})Tr_{x}(a_{i-1})R_{y}(\beta_{i-1})R_{z}(\theta_{i})Tr_{z}(d_{i}),$$

where *i* is the frame number, and the matrix transformations include the rotation matrices corresponding to the twist angle *α_i_*_−1_, the joint angle *θ_i_*, and the rotation parameter *β_i_*_−1_, along with the translation matrices corresponding to the link length *a_i_*_−1_ and the link offset *d_i_*. The tool and base frame transformation matrices, $T_{T}^{6}(\chi_{T})$ and $T_{0}^{W}(\chi_{B})$, respectively, are calculated as:

(2) $$T(\chi )=Tr(x,y,z)R_{z}(\alpha )R_{y}(\beta )R_{x}(\gamma ).$$

The tool frame is calculated with respect to the robot flange and the base frame is calculated with respect to the world frame. The tool and base reference frame parameters are shown in Table 5.

A total of 31 independent parameter errors that are to be identified are included in the complete robot model. The error parameters include 26 kinematic parameters and 5 joint stiffness parameters (*c*_2_, *c*_3_, …, *c*_6_) as listed in Table 6 and Table 7. Indeed, several possible errors are not identified due to redundancies. The first link errors (*δα*_0_, *δa*_0_, *δd*_1_, *δθ*_offs1_) are dependent on the base frame. Furthermore, the axes of joints 2 and 3 are parallel, so only one of either *δd*_2_ or *δd*_3_ should be included model. Finally, since the tool reference frame is precisely measured on a CMM, we do not consider the tool frame parameters into the identification process. Thus, the 31 independent parameters retained are the 6 errors in the coordinates of the base frame, the 19 errors of the MDH parameters of frames 2 to 6 except for *δd*_2_, the skewness parameters between the axes of joints 2 and 3, and the 5 stiffness parameters for joints 2 to 6.

The gearbox of each joint is modeled iteratively in order to identify the stiffness parameters. The gearbox model includes a linear torsional spring coefficient along with the external torque applied to each robot joint, except for the stiffness model of the first joint which is ignored, because of no external torque is applied on joint 1 in our setup. The Newton–Euler [25] algorithm was applied in order to find the external torque of the joints.

With a given set of joint angles, and the initial known parameters, the homogeneous transformation matrix can be calculated with forward kinematic calculations through each frame transformation matrix.

The constant parameter vector of the complete robot model includes all MDH parameters (link twists α, link lengths **a**, link offsets **d** and joint offsets $\theta_{\mathrm{offs}}$), all six stiffness coefficients (**c**) and all base ($\chi_{B}$) and tool frame ($\chi_{T}$) parameters.

(3) $$\rho ={\left[\alpha^{T},a^{T},d^{T},\theta_{\mathrm{offs}}^{T},\beta_{2},\chi_{T}^{T},\chi_{B}^{T},c^{T}\right]}^{T}.$$

Variable parameters are considered to be the input joint configuration vector given as,

(4) $$q={\left[q_{1},q_{2},\ldots ,q_{6}\right]}^{T}$$

and the torques applied to the joint gearbox (**τ**). The kinematic chain of the robot is calculated through consecutive link transformations:

(5) $$T_{6}^{0}(\rho ,q,\tau )=T_{1}^{0}(q_{1})T_{2}^{1}(q_{2})T_{3}^{2}(q_{3})T_{4}^{3}(q_{4})T_{5}^{4}(q_{5})T_{6}^{5}(q_{6}).$$

The transformation chain is completed through implementing the world and tool frames. The final version of the transformation matrix is calculated as,

(6) $$T_{T}^{W}(\rho ,q,\tau )=T_{0}^{W}T_{6}^{0}T_{T}^{6}.$$

The homogeneous matrix transformation is calculated as the estimated position through

(7) $$x_{est}=\left[\begin{array}{c} x_{T}^{W}\\ y_{T}^{W}\\ z_{T}^{W} \end{array}\right]=f(\rho ,q,\tau ).$$

The difference between the measured position data and the estimated position calculations gives the absolute value of the error parameter vector:

(8) $$\Delta x=x_{measured}^{n}-x_{est}^{n}.$$

Identification of the new robot parameters starts with obtaining the position Jacobian matrix, **J**, by differentiating the error vector equations around the robot nominal parameters as,

(9) $$J=\left[\begin{array}{cccc} \frac{\partial f_{1}}{\partial \rho_{1}} & \frac{\partial f_{1}}{\partial \rho_{2}} & \cdots & \frac{\partial f_{1}}{\partial \rho_{m}}\\ \frac{\partial f_{2}}{\partial \rho_{1}} & \frac{\partial f_{2}}{\partial \rho_{2}} & \cdots & \frac{\partial f_{2}}{\partial \rho_{m}}\\ \frac{\partial f_{3}}{\partial \rho_{1}} & \frac{\partial f_{3}}{\partial \rho_{2}} & \cdots & \frac{\partial f_{3}}{\partial \rho_{m}} \end{array}\right],$$

where $\rho_{1},\;\rho_{2},\ldots ,\rho_{m}$ are the 31 independent elements of vector ρ. Let $\overset{⌣}{\rho }={\left[\rho_{1},\rho_{2},\ldots ,\rho_{m}\right]}^{T}$.

For *n* robot measurements, the linearized equation of the position error vector ∆x is given by multiplying the Jacobian matrix with the deviation of the calibration parameters:

(10) $$\Delta x=\frac{\partial x}{\partial \rho }\Delta \overset{⌣}{\rho }=J\Delta \overset{⌣}{\rho }.$$

In our case, the parameter deviation $\Delta \overset{⌣}{\rho }.$ must be the output of the equations since we know the position error vector ∆**x** and the Jacobian matrix elements. The Jacobian matrix is inverted with Moore–Penrose inverse and the equation becomes,

(11) $$\Delta \overset{⌣}{\rho }={\left(J^{T}J\right)}^{-1}J^{T}\Delta x,$$

leaving the deviation parameter vector as the result of the equation

(12) $$\Delta \overset{⌣}{\rho }=J^{+}\Delta x.$$

An observability estimation is made, in order to find the optimal set of joint angles for parameter identification. The aim of using an observability index is to reach better identification in the non-kinematic parameters. Observability index O1 [27] was used, where a previous study showed better results comparing to other observability index equations [28]. The equation is presented as,

(13) $$O_{1}=\frac{{\left(\sigma_{1}\sigma_{2}\sigma_{3}\ldots \sigma_{m}\right)}^{\frac{1}{m}}}{\sqrt{n}},$$

where *n* is the number of joint sets used in measurements, *m* is the number of calibration parameters (31 in our case), *σ* are the singular values taken from the Jacobian matrix. The output joint angles are chosen using the DETMAX algorithm [29]. The algorithm consists of rearranging an initial set of *n* joint angles by adding from and extracting to the *N* configuration pool, until getting the best observability index inside the *n* joint set.

## 6. Validation and Results

Initially, a large pool of feasible robot joint sets (attainable without collisions) corresponding to datum ball probing was generated according to the measurement setup. By using the observability index, 80 of these joint sets were selected for the actual measurements, 20 for each datum ball.

**Figure 8:**
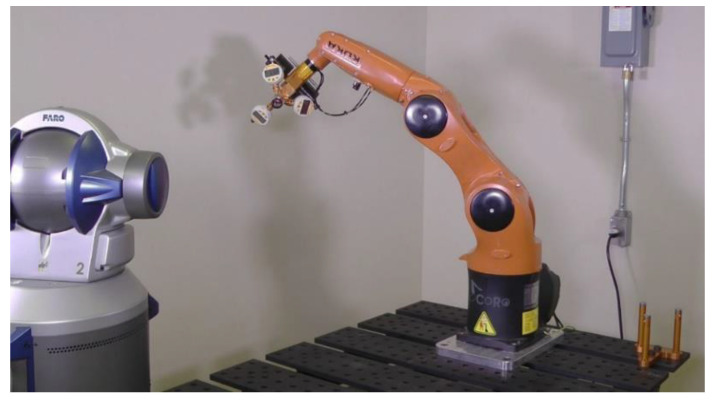
Validation of the robot accuracy in 500 joint sets with a FARO laser tracker.

Once the 31 error parameters are identified, a validation is performed using a FARO laser tracker (Figure 8). Firstly, the world reference frame with respect to the laser tracker frame is obtained by measuring the positions of the three magnetic nests on the 3D ball artifact. Then, the artifact is removed, and an SMR is positioned in the trihedral socket of TriCal with the help of a rare-Earth magnet. Recall that the center of the SMR corresponds exactly to the robot TCP. The robot is then sent to 500 random joint sets, with the only constraints of avoiding collisions and having the SMR face the laser tracker.

The measurements for identification with the TriCal and the measurements for validation with the FARO laser tracker are demonstrated in the *Novel, affordable device for industrial robot calibration* video found on YouTube [30].

**Figure 9:**
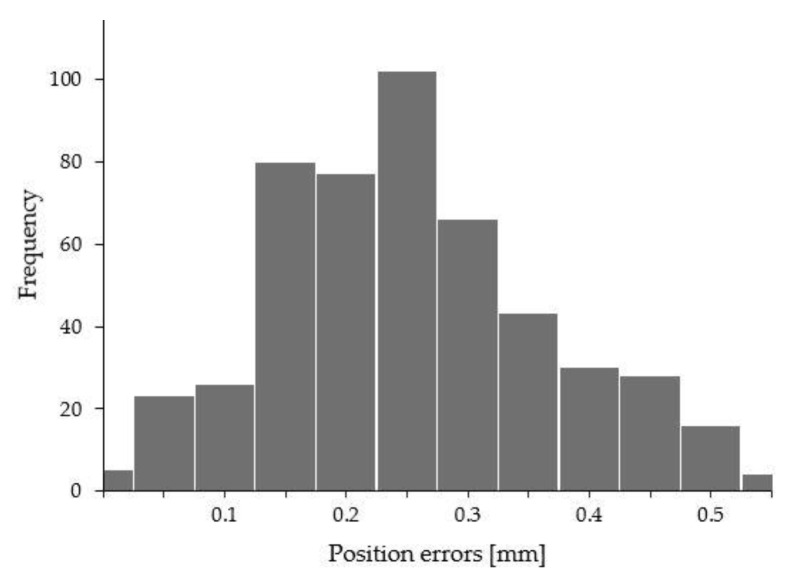
Summary and histogram of position errors after calibration with TriCal.

The robot parameters as identified after measurements with the TriCal are presented in Table 8. Then the position errors after calibration (i.e., after using the identified parameters in Table 8) and measured with the laser tracker in 500 poses are presented in Figure 9.

Finally, in order to compare the efficiency of the TriCal to that of the laser tracker, when it comes to robot calibration, we also performed an identification using measurements taken only with a laser tracker. Specifically, we took 80 measurements throughout the workspace of the robot, selected using the observability analysis described in Section 5. Then, the accuracy after calibration was measured in the same 500 poses used in the case of TriCal.

The final results are shown in Table 9. Note that in all tests, the weight of the end-effector was 6 kg, i.e., the maximum rated payload of the robot. As expected, the laser tracker leads to slightly better results, because the measurements used for identification are more evenly distributed throughout the workspace of the robot.

## 7. Conclusions

A practical method for on-site robot calibration based on a novel wireless 3D measurement device was presented. The method was tested on a KUKA KR6 R700 sixx industrial robot. It was found that the efficiency of the new method is close to that of a laser tracker, when it comes to calibrating a small industrial robot. To further improved the efficiency of our method, more research is needed on the design of the 3D ball artifact. A wider distribution of the datum balls is needed, and the artifact has to be more portable.

That said, the new device and the 3D ball artifact are several times less expensive than the most affordable laser tracker. Indeed, the cost of the new device is less than USD 5000. Furthermore, although not discussed in this paper, we may argue that our device is better suited to characterizing the repeatability of an industrial robot, as it is more precise at measuring micrometer displacements.

## Figures and Tables

**Table 1:** Positions of the four 1-in datum balls with respect to the world reference frame

Ball	x [mm]	y [mm]	z [mm]
1	−79.539	−330.141	17.531
2	−79.193	287.805	18.740
3	−79.650	−21.958	276.388
4	26.379	−21.676	170.489

**Table 2:** Proposed Calibration Procedure.

Proposed Calibration Procedure
Attach 3D ball artifact, preliminarily inspected on a CMM, to the robot’s base support, in a precisely known location. Attach TriCal, preliminarily adjusted with the desired total weight and inspected on a CMM, to the flange of the industrial robot. Start robot in manual mode, reorient TriCal upwards and then manually master the three digital indicators. Switch robot to automatic mode and run RoboDK on PC connected to the robot. Execute Python code for probing each of the four datum balls with 20 different orientations, preliminarily determined through observability analyses. Each probing is based on the auto-centering procedure outlined in Figure 5. Once all 80 joint targets are recorded, identify the 31 robot parameters. Once these 31 robot parameters identified, use RoboDK to calculate so-called fake targets for each new desired end-effector pose.

**Table 3:** Procedure for measuring the precision of TriCal.

Proposed Procedure (Refer to Figure 7)
Place the 1.5-in master ball in the trihedral socket of the triaxial mount. Master all three digital indicators. Measure the position of the master ball with the CMM. Remove the master ball. Automatically position a 1-in datum ball attached to a highly precise six-axis robot until all three indicators are at zero. Measure the position of the datum ball with the CMM. Move the datum ball away. Repeat step 5–7 two more times.

**Table 4:** KUKA KR6 R700 Nominal Modified Denavit–Hartenberg (MDH) Parameters.

*i*	$\alpha_{i-1}$ [°]	$a_{i-1}$ [mm]	$d_{i}$ [mm]	$\theta_{i}$ [°]	$\beta_{i-1}$ [°]
1	180	0	−400	$\theta_{1}$	n/a
2	90	25	0	$\theta_{2}$	n/a
3	0	315	0	$\theta_{3}-90$	0
4	90	35	−365	$\theta_{4}$	n/a
5	−90	0	0	$\theta_{5}$	n/a
6	90	0	−80	$\theta_{6}$	n/a

**Table 5:** Base and Tool Nominal Parameters.

Frame	*x* [mm]	*y* [mm]	*z* [mm]	*α* [°]	*β* [°]	*γ* [°]
Base	$x_{0}^{W}$	$y_{0}^{W}$	$z_{0}^{W}$	$\alpha_{0}^{W}$	$\beta_{0}^{W}$	$\gamma_{0}^{W}$
Tool	$x_{T}^{6}$	$y_{T}^{6}$	$z_{T}^{6}$	$\alpha_{T}^{6}$	$\beta_{T}^{6}$	$\gamma_{T}^{6}$

**Table 6:** Base and Tool Calibration Parameters.

Frame	*x* [mm]	*y* [mm]	*z* [mm]	*α* [°]	*β* [°]	*γ* [°]
Base	$x_{0}^{W}+\delta x_{0}^{W}$	$y_{0}^{W}+\delta y_{0}^{W}$	$z_{0}^{W}+\delta z_{0}^{W}$	$\alpha_{0}^{W}+\delta \alpha_{0}^{W}$	$\beta_{0}^{W}+\delta \beta_{0}^{W}$	$\gamma_{0}^{W}+\delta \gamma_{0}^{W}$
Tool	$x_{T}^{6}$	$y_{T}^{6}$	$z_{T}^{6}$	$\alpha_{T}^{6}$	$\beta_{T}^{6}$	$\gamma_{T}^{6}$

**Table 7:** Robot Calibration Kinematic and Joint Stiffness Parameters.

*i*	$\alpha_{i-1}$ [°]	$a_{i-1}$ [mm]	$d_{i}$ [mm]	$\theta_{i}$ [°]	$\beta_{i-1}$ [°]
1	$\alpha_{0}$	$a_{0}$	$d_{1}$	$q_{1}+\theta_{offs,1}$	n/a
2	$\alpha_{1}+\delta \alpha_{1}$	$a_{1}+\delta a_{1}$	$d_{2}$	$q_{2}+\theta_{offs,2}+\delta \theta_{offs,2}+c_{2}\tau_{2}$	n/a
3	$\alpha_{2}+\delta \alpha_{2}$	$a_{2}+\delta a_{2}$	$d_{3}+\delta d_{3}$	$q_{3}+\theta_{offs,3}+\delta \theta_{offs,3}+c_{3}\tau_{3}$	$\beta_{2}+\delta \beta_{2}$
4	$\alpha_{3}+\delta \alpha_{3}$	$a_{3}+\delta a_{3}$	$d_{4}+\delta d_{4}$	$q_{4}+\theta_{offs,4}+\delta \theta_{offs,4}+c_{4}\tau_{4}$	n/a
5	$\alpha_{4}+\delta \alpha_{4}$	$a_{4}+\delta a_{4}$	$d_{5}+\delta d_{5}$	$q_{5}+\theta_{offs,5}+\delta \theta_{offs,5}+c_{5}\tau_{5}$	n/a
6	$\alpha_{5}+\delta \alpha_{5}$	$a_{5}+\delta a_{5}$	$d_{6}+\delta d_{6}$	$q_{6}+\theta_{offs,6}+\delta \theta_{offs,6}+c_{6}\tau_{6}$	n/a

**Table 8:** Comparison of nominal and identified robot calibration parameters.

Parameters	Nominal	Identified
$x_{0}^{W}$ [mm]	−536.320	−538.014
$y_{0}^{W}$ [mm]	−21.901	−23.563
$z_{0}^{W}$ [mm]	−58.349	−58.157
$\alpha_{0}^{W}$ [°]	0.000	0.254
$\beta_{0}^{W}$ [°]	0.000	0.014
$\gamma_{0}^{W}$ [°]	0.000	0.172
$\alpha_{1}$ [°]	90.000	90.005
$\alpha_{2}$ [°]	0.000	−0.015
$\alpha_{3}$ [°]	90.000	89.999
$\alpha_{4}$ [°]	−90.000	−89.895
$\alpha_{5}$ [°]	90.000	89.983
$a_{1}$ [mm]	25.000	25.243
$a_{2}$ [mm]	315.000	315.16
$a_{3}$ [mm]	35.000	35.106
$a_{4}$ [mm]	0.000	0.075
$a_{5}$ [mm]	0.000	0.032
$d_{3}$ [mm]	0.000	0.067
$d_{4}$ [mm]	−365.000	−365.34
$d_{5}$ [mm]	0.000	0.001
$d_{6}$ [mm]	−80.000	−80.279
$\theta_{offs,2}$ [°]	0.000	0.026
$\theta_{offs,3}$ [°]	−90.000	−89.956
$\theta_{offs,4}$ [°]	0.000	0.007
$\theta_{offs,5}$ [°]	0.000	0.015
$\theta_{offs,\;6}$ [°]	0.000	0.012
$\beta_{2}$ [°]	0.000	−0.005
$c_{2}\;[{}^{\circ}/\mathrm{Nm}\times 10^{-3}]$	0.000	2.987
$c_{3}\;[{}^{\circ}/\mathrm{Nm}\times 10^{-3}]$	0.000	−3.238
$c_{4}\;[{}^{\circ}/\mathrm{Nm}\times 10^{-3}]$	0.000	−8.723
$c_{5}\;[{}^{\circ}/\mathrm{Nm}\times 10^{-3}]$	0.000	−25.489
$c_{6}\;[{}^{\circ}/\mathrm{Nm}\times 10^{-3}]$	0.000	−28.161

**Table 9:** Comparison of position errors after calibration with TriCal and laser tracker.

Method	Mean [mm]	Maximum [mm]	Standard Deviation [mm]
TriCal	0.326	0.624	0.107
Laser Tracker	0.231	0.539	0.087
